# *Staphylococcus epidermidis* in Orthopedic Device Infections: The Role of Bacterial Internalization in Human Osteoblasts and Biofilm Formation

**DOI:** 10.1371/journal.pone.0067240

**Published:** 2013-06-28

**Authors:** Florent Valour, Sophie Trouillet-Assant, Jean-Philippe Rasigade, Sébastien Lustig, Emmanuel Chanard, Hélène Meugnier, Sylvestre Tigaud, François Vandenesch, Jérome Etienne, Tristan Ferry, Frédéric Laurent, Lyon BJI Study Group

**Affiliations:** 1 INSERM U1111, International Center for Research in Infectiology, Lyon, France; 2 Laboratory of Bacteriology, North Lyon University Hospital, Hospices Civils de Lyon, Lyon, France; 3 Infectious Diseases Department, Hospices Civils de Lyon, Lyon, France; 4 Orthopedic Surgery Department, Hospices Civils de Lyon, Lyon, France; 5 Laboratory of Bacteriology, Novecia, Lyon, France; 6 Laboratory of Bacteriology, East Lyon University Hospital, Hospices Civils de Lyon, Lyon, France; 7 Claude Bernard University, Lyon, France; 8 French National Reference Center for Staphylococci, Lyon, France; Charité-University Medicine Berlin, Germany

## Abstract

**Background:**

*Staphylococcus epidermidis* orthopedic device infections are caused by direct inoculation of commensal flora during surgery and remain rare, although *S. epidermidis* carriage is likely universal. We wondered whether *S. epidermidis* orthopedic device infection strains might constitute a sub-population of commensal isolates with specific virulence ability. Biofilm formation and invasion of osteoblasts by *S. aureus* contribute to bone and joint infection recurrence by protecting bacteria from the host-immune system and most antibiotics. We aimed to determine whether *S. epidermidis* orthopedic device infection isolates could be distinguished from commensal strains by their ability to invade osteoblasts and form biofilms.

**Materials and Methods:**

Orthopedic device infection *S. epidermidis* strains (n = 15) were compared to nasal carriage isolates (n = 22). Osteoblast invasion was evaluated in an *ex vivo* infection model using MG63 osteoblastic cells co-cultured for 2 hours with bacteria. Adhesion of *S. epidermidis* to osteoblasts was explored by a flow cytometric approach, and internalized bacteria were quantified by plating cell lysates after selective killing of extra-cellular bacteria with gentamicin. Early and mature biofilm formations were evaluated by a crystal violet microtitration plate assay and the Biofilm Ring Test method.

**Results:**

No difference was observed between commensal and infective strains in their ability to invade osteoblasts (internalization rate 308+/−631 and 347+/−431 CFU/well, respectively). This low internalization rate correlated with a low ability to adhere to osteoblasts. No difference was observed for biofilm formation between the two groups.

**Conclusion:**

Osteoblast invasion and biofilm formation levels failed to distinguish *S. epidermidis* orthopedic device infection strains from commensal isolates. This study provides the first assessment of the interaction between *S. epidermidis* strains isolated from orthopedic device infections and osteoblasts, and suggests that bone cell invasion is not a major pathophysiological mechanism in *S. epidermidis* orthopedic device infections, contrary to what is observed for *S. aureus*.

## Introduction

*Staphylococcus epidermidis*, coagulase-negative staphylococci, have been considered innocuous commensals of human skin and mucous membranes but are now accepted as the leading opportunistic pathogens responsible for numerous nosocomial infections [1]. In particular, they account for 30 to 43% of joint prosthesis infections [2]. The current accepted pathophysiological mechanism of *S. epidermidis* orthopedic device infection is the direct inoculation of skin colonizing strains at the time of surgery [2]–[4].

The contrast between the low incidence of *S. epidermidis* orthopedic device infection and the highly prevalent *S. epidermidis* carriage suggests that *S. epidermidis* bone and joint infections might either correspond to accidental events due to colonizing strains or to a specific, more virulent sub-population of commensal isolates. The existence of such specificity could be crucial because it may impact the prevention and management of these severe infections. The ability of a commensal pathogen to cause chronic diseases that are difficult to treat suggests that these infections are attributable to the ability of this organism to escape the immune system and antibiotic therapy [1], [5].

Two predominant mechanisms have been proposed to be implicated in orthopedic device infections: i) bacterial invasion and persistence within non-professional phagocytes such as osteoblasts, as previously demonstrated for *S. aureus* [6], and ii) the ability of bacteria to form biofilms [7]–[10]. The aim of the present study was to determine if *S. epidermidis* strains implicated in orthopedic device bone and joint infections can be distinguished from colonizing ones on the basis of these proposed mechanisms. We assayed 22 nasal carriage *S. epidermidis* strains and 15 bone-infective isolates in *ex vivo* models of human osteoblast infection and biofilm formation assays. This study received the approval of the French South-East ethics committee.

## Materials and Methods

### Ethical Statement

This study received the approval of the French South-East ethics committee, with the reference number 2013-018. In accordance with the French legislation, written informed patient consent was not required for any part of the study, and especially for the human primary osteoblasts collection.

### Bacterial Strains

We analyzed all *S. epidermidis*-positive bacteriological samples collected in the orthopedic department of our institution between 2001 and 2011. Twenty-four strains that contributed to monomicrobial orthopedic device infections (i.e., clinical evidence of infection and at least 2 deep bacteriological *S. epidermidis*-positive samples [2]) were considered for inclusion. For technical reasons, gentamicin-resistant strains were excluded (n = 9); thus, 15 clinical orthopedic device infection isolates were included in the study (total hip prosthesis (n = 5), total knee prosthesis (n = 6), intramedullar osteosynthesis device (n = 4)). Thirty-three *S. epidermidis* colonizing isolates were selected from the staphylococcal strains collected from nasal swabs of patients at admission in the orthopedic surgery unit from June to December 2010. After exclusion of gentamicin-resistant strains, 22 commensal isolates were analyzed in this study. Identification of all *S. epidermidis* isolates was confirmed by matrix-assisted laser desorption ionization time-of-flight mass spectrometry (MALDI-TOF-MS) as previously described [11].

The nasal colonizing *S. epidermidis* NCTC11047 strain, previously evaluated in a similar bone cell infection model [12], was used as a control in each experiment. The *S. aureus* 8325-4 laboratory strain, which has been well characterized in its ability to invade osteoblasts [13], was used as a control in each osteoblast infection experiment. Prior to performing the assays, *S. epidermis* cells were grown overnight (18 h) in a brain heart infusion (BHI) (AES, Bruz, France) aerobically at 36.5°C. Five hundred microliters of this suspension were transferred in a sterile BHI and grown for 3 h aerobically at 36.5°C to obtain log-phase cultures. Bacteria were harvested by centrifugation and suspended in an assay medium. Suspensions were standardized on the basis of optical density at 600 nm (OD_600_) using a photometric regression formula established in preliminary experiments (data not shown): CFU/mL  = 7. 10^8^×OD_600_–10^8^ for *S. epidermidis* and CFU/mL  = 7.10^8^×OD_600_–3.10^7^ for *S. aureus*.

### Human Osteoblast Culture

All cell culture reagents were obtained from GIBCO (Paisley, United Kingdom). The human osteoblastic cell line MG63 (CRL-1427), which was purchased from LGC standard (USA), was routinely cultured in growth medium consisting of Dulbecco’s modified Eagle’s medium (DMEM) supplemented with 10% fetal calf serum (FBS) and contained 25 mM HEPES, 2 mM L-glutamine, 100 U/mL penicillin, and 100 µg/mL streptomycin (“growth medium with antibiotics”). Cells were passaged twice a week and were used in experiments up to passage 20 after being thawed from a stock culture.

Primary human osteoblasts were isolated from non-infected patients and sampled during routine hip surgery. Bone specimens were collected on the day of surgery in phosphate-buffered saline (PBS, bioMérieux, Marcy l’Etoile, France) containing penicillin (100 U/mL) and streptomycin (100 µg/mL) and processed the same day. Excess blood and soft tissue were removed with a scalpel aided by several PBS rinses. Samples were cut into fragments of 1 to 2 mm^3^ and cultured in routine growth medium with antibiotics and supplemented with 50 µg/mL of ascorbic acid (Sigma Aldrich, St Louis, USA). The medium was thereafter changed once a week until cells became confluent. Osteoblasts were characterized by an osteocalcin assay, frozen, and used only once upon thawing.

### Invasion of Osteoblasts by S. epidermidis Using a Gentamicin Protection Assay (Fig. 1)

Osteoblasts were seeded at 40,000 cells per well into 24-well tissue cultures plates (Falcon, Le Pont de Claix, France) in 1 mL of growth medium with antibiotics and cultured for 2 days until 70 to 80% confluence. Cells were washed twice with 1 mL of DMEM before the addition of bacteria. Bacterial suspensions in growth medium without antibiotics were added to the bone cell culture wells at a multiplicity of infection (MOI) of 500∶1 for *S. epidermidis* and 100∶1 for *S. aureus*. After 2 h of co-culture of bacteria and osteoblasts in a 37°C/5% CO_2_ incubator, cell cultures were washed twice with 1 mL DMEM and incubated 1 hour with growth medium supplemented with 200 µg/mL gentamicin (PAA, Pasching, Austria) to kill the remaining extracellular staphylococcal cells. One hundred microliters of each culture supernatant were plated onto tryptic soy agar plates (TSA, Oxoid, Dardilly, France) to confirm the absence of living extracellular bacteria. Osteoblast cultures were washed twice with DMEM and subsequently lysed by a 10-minute incubation period with sterile purified water. To quantify internalized bacteria, suspension dilutions of cell lysates were plated in duplicate on TSA plates followed by an overnight incubation at 37°C.

**Figure 1:**
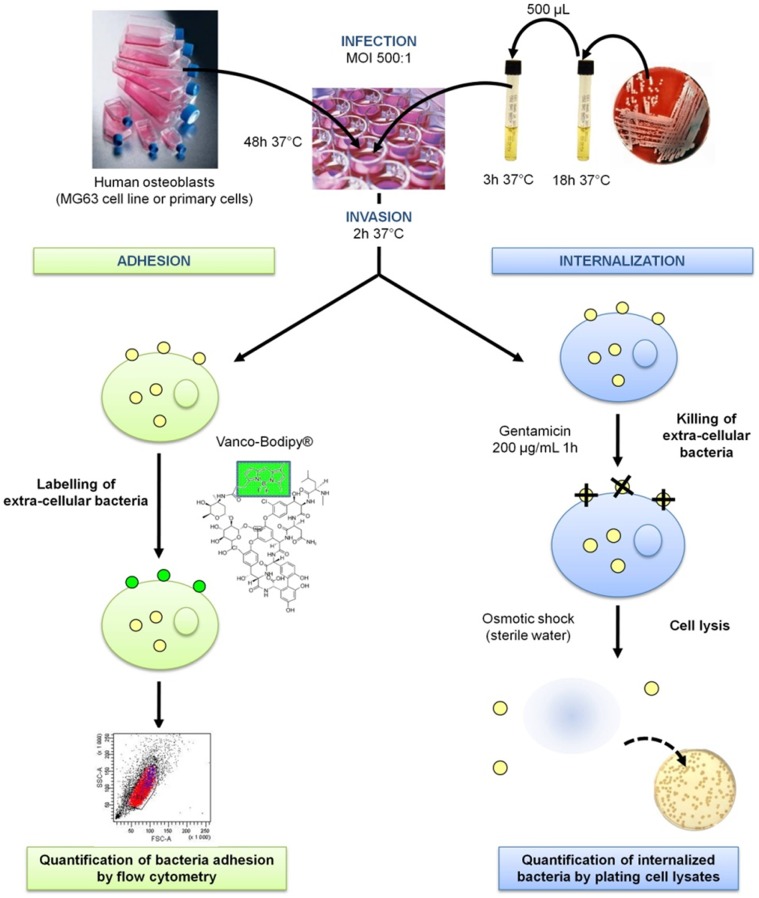
Gentamicin protection assay. MOI: Multiplicity of infection.

### Adhesion of S. epidermidis to Osteoblasts (Fig. 1)

*Staphylococcus epidermidis* adhesion to osteoblasts was assessed using a flow cytometry-based assay as previously described [13]. Three colonizing and three infective *S. epidermidis* strains were randomly selected and tested in addition to *S. epidermidis* NCTC11047 and *S. aureus* 8325-4 strains, which were used as controls. Briefly, after 2 h of bacterial co-culture (MOI of 500∶1 and 100∶1 for *S. epidermidis* and *S. aureus*, respectively), osteoblasts were washed twice with DMEM, harvested from the plates using a 0.05% trypsin-EDTA (GIBCO, Paisley, United Kingdom) treatment for 2 min at 37°C, and washed twice with ice-cold PBS. Then, they were incubated on ice and in the dark for 15 min with a 1∶1 mixture of vancomycin and BODIPY FL vancomycin (Invitrogen, Grand Island, USA) at a concentration of 0.8 µg/mL, which targets the bacterial peptidoglycan and cannot penetrate intact cells. After labeling, cells were washed twice with PBS and fixed in 1% formaldehyde (Sigma Aldrich, St Louis, USA). Flow cytometry analysis was performed with a Canto II cytometer (Becton Dickinson) using the FL-1 channel to measure the fluorescence intensity and the FSC/SCC stopping gate to exclude cellular debris and unbound bacteria. The fluorescence intensity marker (M) was set to include less than 2% of uninfected cells (negative control). The total number of bacteria was estimated as the mean fluorescence intensity of cells in M multiplied by the proportion of cells in M, which is represented in arbitrary fluorescence units (AFU) [13].

### S. epidermidis Biofilm Formation

Biofilm formation was evaluated by a tissue culture plate method using crystal violet, which allows for a semi-quantitative measurement of mature biofilms [14]. After adjusting the OD_600_ of overnight bacterial suspensions at 1+/−0.05, they were diluted 1∶100 in sterile BHI, and 100 µL of bacterial suspension were seeded in 96-well flat bottom plates (Becton Dickinson). Four wells were used for each isolate. Negative control wells contained BHI alone.

After 24 and 48 h of growth at 37°C, the contents of each well were gently removed, and the wells were washed three times with water to suppress the free-floating bacteria. Biofilms formed by adherent bacteria were fixed with 100 µL of 99% methanol (VWR International, Briare, France) per well for 20 min and air dried for 1 h under a chemical hood. Fixed bacteria were colored with 100 µL of 0.1% crystal violet (Merck, Fontenay-sous-bois, France) per well for 10 min, and excess stain was rinsed off by washing five times with water before air-drying. Dye bound to the biofilm was resolubilized with 100 µl of 33% acetic acid (VWR International) per well. The OD_490_ was measured in each well with a micro ELISA Auto Reader, Model 680 (BioRad, Hercules, USA). Tests were performed in triplicate, and an average OD_490_ value was calculated for each strain and for the negative controls. Finally, the OD_490_ value of the tested strain was expressed as an average OD_490_ value of the tested strain normalized to the control OD_490_ value.

The ability to form biofilms was also explored using the BioFilm Ring test (BioFilm Control, Saint Beauzire, France), as described by Chavant et al [15]. Briefly, this technique includes the immobilization of magnetic beads embedded in bacterial aggregates following biofilm formation. After adjusting the OD_600_ of overnight bacterial suspensions at 1+/−0.05, the suspensions were diluted 1∶250 in fresh BHI and mixed with 1% of tonner solution corresponding to a magnetic bead suspension. The mixtures were homogenized, and 200 µl were deposited in each well of a 96-well microplate (12 8-well polystyrene strips). Each strain was cultured in duplicate. Every 2 h for 24 h, wells of one plate were covered with 100 µl of contrast solution (white opaque oil). The entire strip was placed for 1 min on a dedicated block test (magnet support) for magnetization contact and scanned with the plate reader to obtain an image for each well. During magnetization contact, free beads were attracted to the center of the bottom of wells and formed a spot, but beads blocked within the biofilm remain in suspension. The BioFilm Control software (BioFilm Elements) analyzes the images of each well before and after magnetization and calculates a value termed the BioFilm Index (BFI). Each observed BFI (BFI_o_) was normalized to a proportion of immobilized beads (RBI for Relative Bead Immobilization) compared with controls with (BFI_reference_) and without (BFI_minimal_) beads using the following formula: RBI = [(BFI_reference_-BFI_o_)/(BFI_reference_ – BFI_minimal_) ] ^0.5^.

### Molecular Characterization by Pulsed-field Gel Electrophoresis

*Staphylococcus epidermidis* isolates were submitted to pulsed-field gel electrophoresis (PFGE) analysis to assess their genetic diversity. Genomic DNA was extracted from log-phase cultures grown for 2 h in BHI broth, prepared in low-melting point agarose plugs (InCert agarose, Lonza, Basel, Switzerland) and digested with the endonuclease *Sma*I (Roche, Rosny-sous-bois, France). DNA fragments were resolved on a 1% agarose gel (SeaKem agarose, Lonza, Basel, Switzerland) with a contour-clamped homogeneous electric field apparatus GenePath (BioRad, Marnes-la-coquette, France) at 6 V/cm with switching times ramped from 5 to 35 s for 20 h at 14°C. Gels were stained with Sybr Safe (Invitrogen, Carlsbad, USA). PFGE profiles and phylogenic analysis were performed with the GelCompar II software included in Bionumerics from Applied Maths (Kortrijk, Belgium). A dendrogram was generated using the Pearson correlation coefficient and by clustering using the unweighted pair group method analysis (UPGMA) with average linkages at a maximum position tolerance of 2%. The PFGE profiles were interpreted according to the criteria of Tenover et al. Isolates with DNA fingerprints differing by more than three bands and a similarity of less than 80% provided by the dendrogram analysis were interpreted as different PFGE types.

### Statistical Analysis

Descriptive statistics were used to estimate the frequencies of the study variables, which are described as the means and standard deviations. Differences between colonizing and infective strains were analyzed using the Mann-Whitney U tests, except for the Biofilm Ring test kinetic of biofilm formation, which was assessed by a two-way ANOVA test. A value of p<0.05 was significant. All analyses were performed using XLSTAT v.2011.1.02 (Addinsoft).

## Results

### Internalization of S. epidermidis in Human Osteoblasts

**Figure 2:**
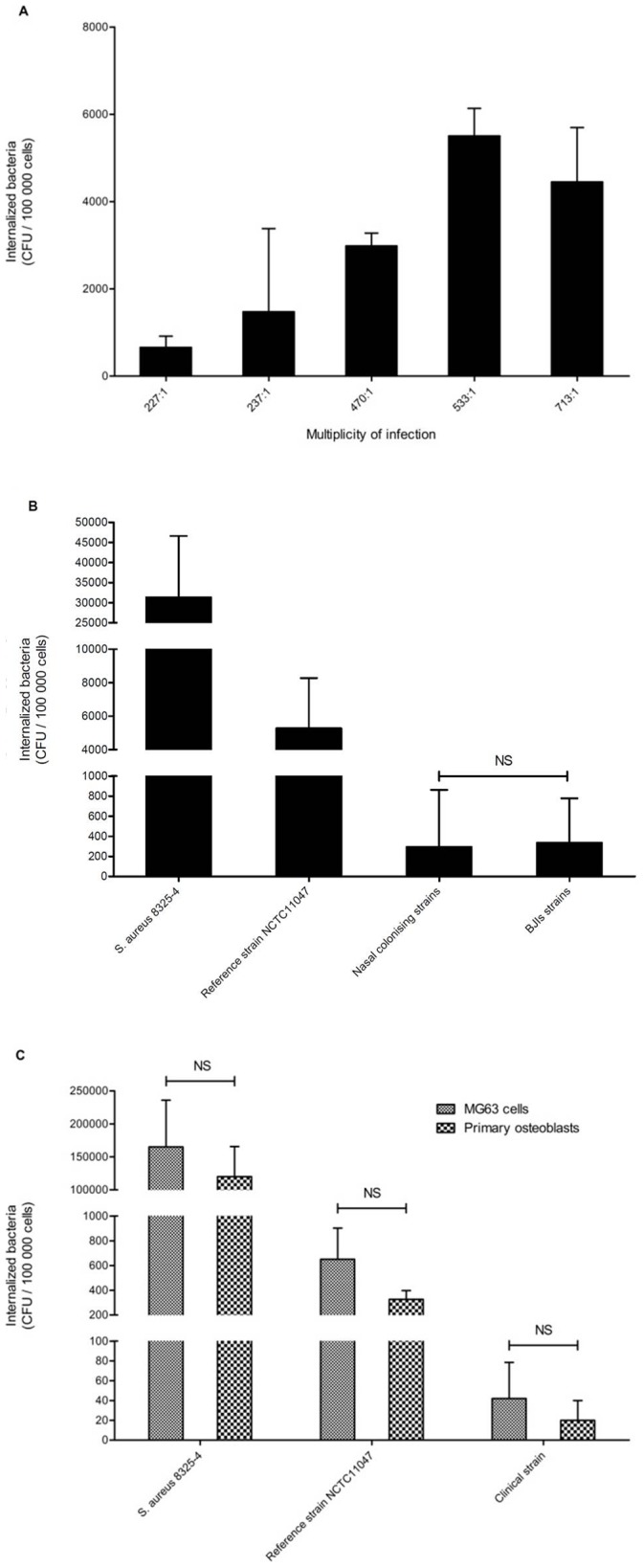
Invasion of human osteoblasts by *S. epidermidis* clinical isolates. A. Determination of the optimal multiplicity of infection (MOI) for use in osteoblast invasion assays (reference strain NCTC11047). B. Evaluation of the rate at which *S. epidermidis* strains invade the MG63 osteoblastic cell line for nasal colonization (n = 22) versus orthopedic infection (n = 15) isolates. C. Comparison of the invasion rate in MG63 osteoblastic cells and primary human osteoblasts for *S. aureus* 8325-4, *S. epidermidis* NCTC11047, and one randomly chosen *S. epidermidis* clinical isolate. Data are represented as the means and standard deviations of three replicate cultures from one gentamicin protection assay. NS: Not significant.

MG63 osteoblasts were infected with increasing MOIs of the reference strain NCTC11047 (fig. 2A), and the results demonstrated that the number of bacteria that are internalized was MOI-dependent, where more *S. epidermidis* was internalized when the MOI rose from 250∶1 to 500∶1. Based on these results, the MOI of 500∶1 was chosen for all further invasion experiments. The ability of each of the clinical *S. epidermidis* strains to invade MG63 osteoblasts was evaluated in triplicate using a gentamicin protection assay. No difference in the osteoblast invasion rate was observed between colonizing and orthopedic device infection strains, where the numbers of internalized bacteria were 308+/−631 and 347+/−431 CFU/well, respectively, corresponding to 0.0008+/−0.0013% and 0.0004+/−0.0005% of the inoculums (p = 0.973) (fig. 2B). The internalization rate of the reference strain *S. epidermidis* NCTC11047 was estimated at 5165+/−2748 CFU/well (0.013+/−0.009% of the inoculums). The internalization rate of *S. aureus* 8325-4 was estimated at 0.37+/−0.20% of the inoculums.

To exclude a bias due to a “cell line effect”, this low internalization rate of *S. epidermidis* in MG63 cells was confirmed using primary bone cells by testing the invasion ability of the reference strain NCTC11047 (0.0018+/−0.002%) and a randomly-chosen clinical strain (0.0001+/−0.0002%) (fig. 2C).

### Adhesion of S. epidermidis to Human Osteoblasts

**Figure 3:**
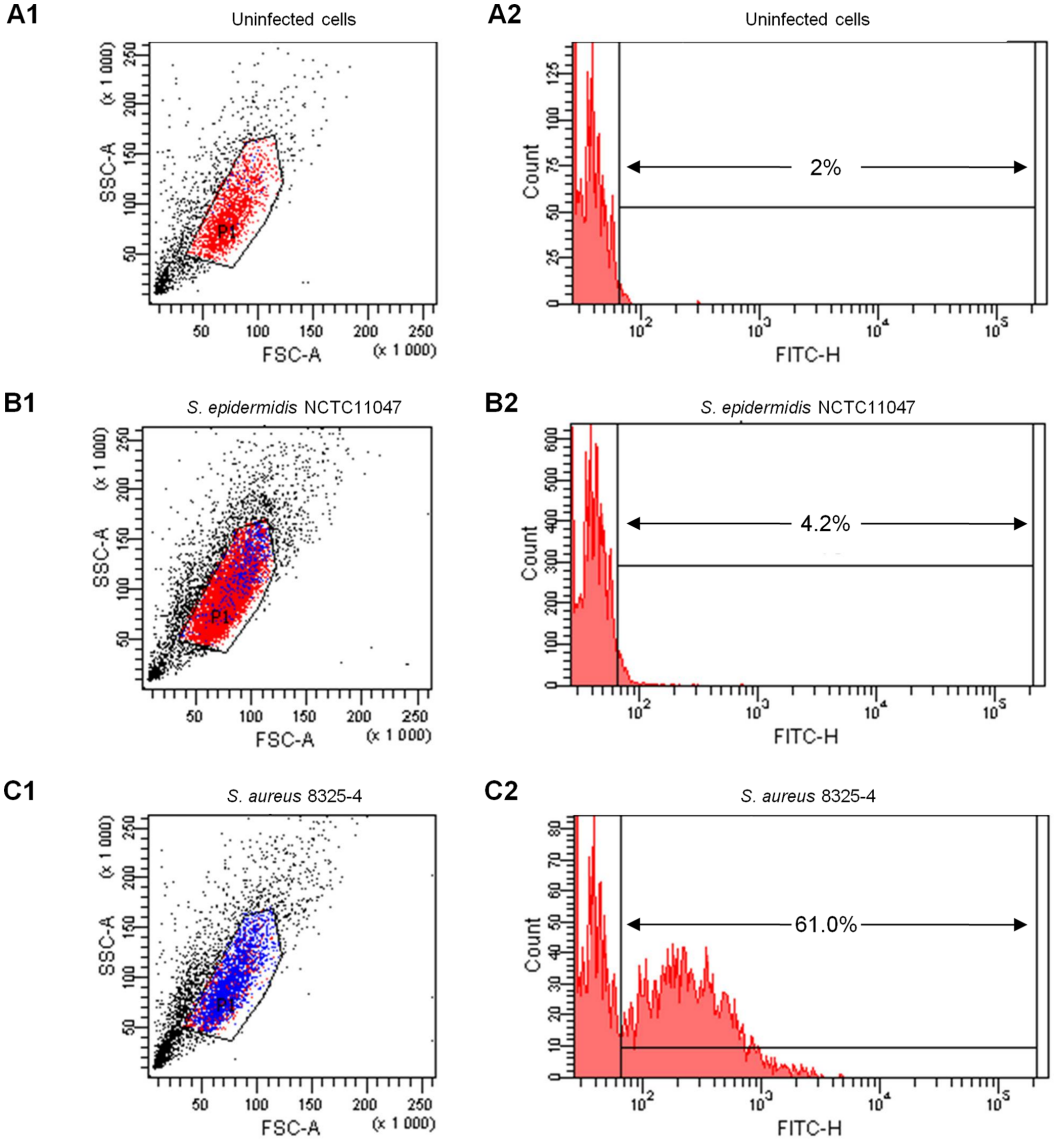
Flow cytometric quantification of adhesion to MG63 osteoblasts by *S. epidermidis*. Flow cytometry analysis was performed using an FSC/SSC stopping gate with uninfected cells (A1), with a fluorescence intensity marker M set to include <2% of uninfected cells (A2). The results from the original cytogram (1) and the distribution of FL1-positive events (2) of a representative experiment are shown, including the reference strains *S. epidermidis* NCTC1147 (B) and *S. aureus* 8325-4 (C).

**Figure 4:**
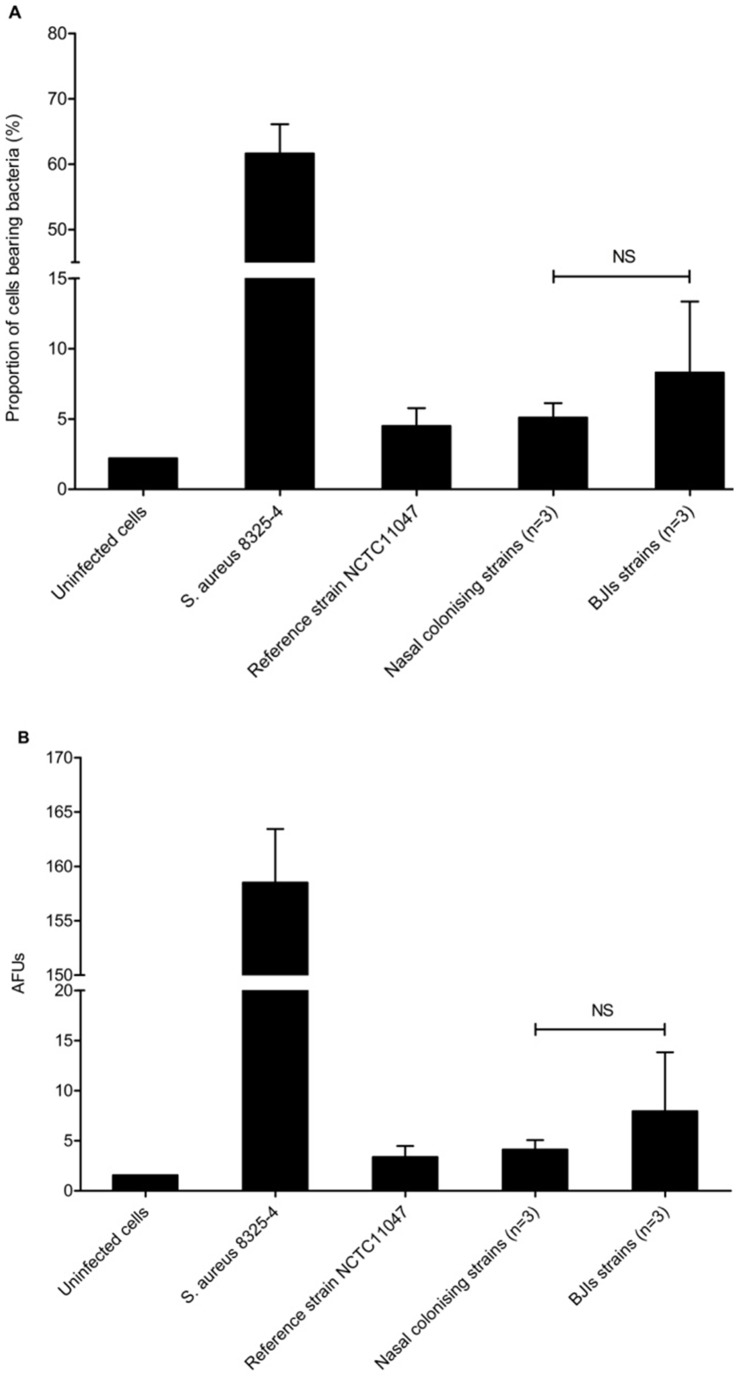
Quantification of *S. epidermidis* adhesion to MG63 osteoblasts. After 2 h of co-culture of MG63 osteoblasts with infective and colonizing *S. epidermidis* strains (n = 3 each) and *S. aureus* strains at an MOI of 500∶1 and 100∶1, respectively, adhered bacteria were labeled using the membrane-impermeable fluorochrome BODIPY FL vancomycin. The results are presented as the means and standard deviations of the proportion of cells bearing bacteria (A) and of arbitrary fluorescence units (AFUs) (B).

Given the low rate of invasion observed for *S. epidermidis* and that adhesion is a prerequisite for internalization, we assessed the ability of six randomly selected *S. epidermidis* clinical strains to adhere to MG63 osteoblasts. *S. epidermidis* NCTC11047 and *S. aureus* 8325-4 were used as controls (fig. 3 and 4). The AFU for the NCTC11047 reference strain and clinical strains were 3.38+/−1.1 and 6.04+/−3.8, respectively, corresponding to 4.5% +/−1.3% and 6.7% +/−3.4% of cells bearing bacteria. No significant differences between nasal colonizing (n = 3) and infective (n = 3) strains were observed (p = 0.513). For comparison, the AFU obtained for *S. aureus* was higher at 158.5+/−4.93, corresponding to 61.7% of cells associated with bacteria. As expected, the background fluorescence control (uninfected cells) was low (AFU  = 1.6).

### Biofilm Formation

**Figure 5:**
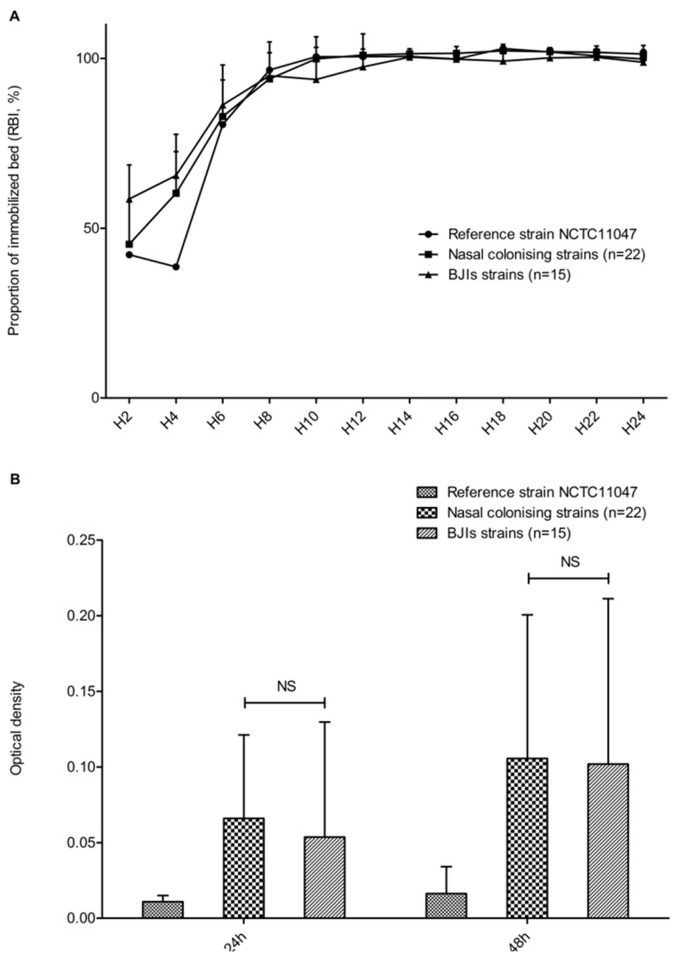
Evaluation of biofilm-formation ability of *S. epidermidis* clinical isolates. A. Kinetics of early biofilm formation was assayed by the Biofilm Ring Test method for the reference strain NCTC11047 and for infective (n = 15) and colonizing (n = 22) *S. epidermidis* strains. B. Quantification of mature biofilm formation after 24 and 48 h by the crystal violet staining method for the *S. epidermidis* reference strain NCTC11047 and 37 clinical isolates.

The Biofilm Ring test was used to assess the early biofilm-forming ability of the clinical *S. epidermidis* strains. No differences were observed between the nasal colonizing and infective strains (two-way ANOVA, p = 0.81), and the results agreed with those observed for the reference strain NCTC11047. All strains produced biofilms and completely immobilized the beads after 8 to 12 hours (fig. 5A). For comparison, in our experimental conditions, *S. aureus* 8325-4 completely immobilized the beads in less than 4 hours as did most of the clinical *S. aureus* isolates (data not shown).

Similarly, the crystal violet staining method revealed no differences in mature biofilm-forming ability between the two groups at 24 h (p = 0.051) and 48 h (p = 0.93) (fig. 5B).

### Genetic Diversity: Pulse-field Gel Electrophoresis (PFGE) Patterns Analysis

**Figure 6:**
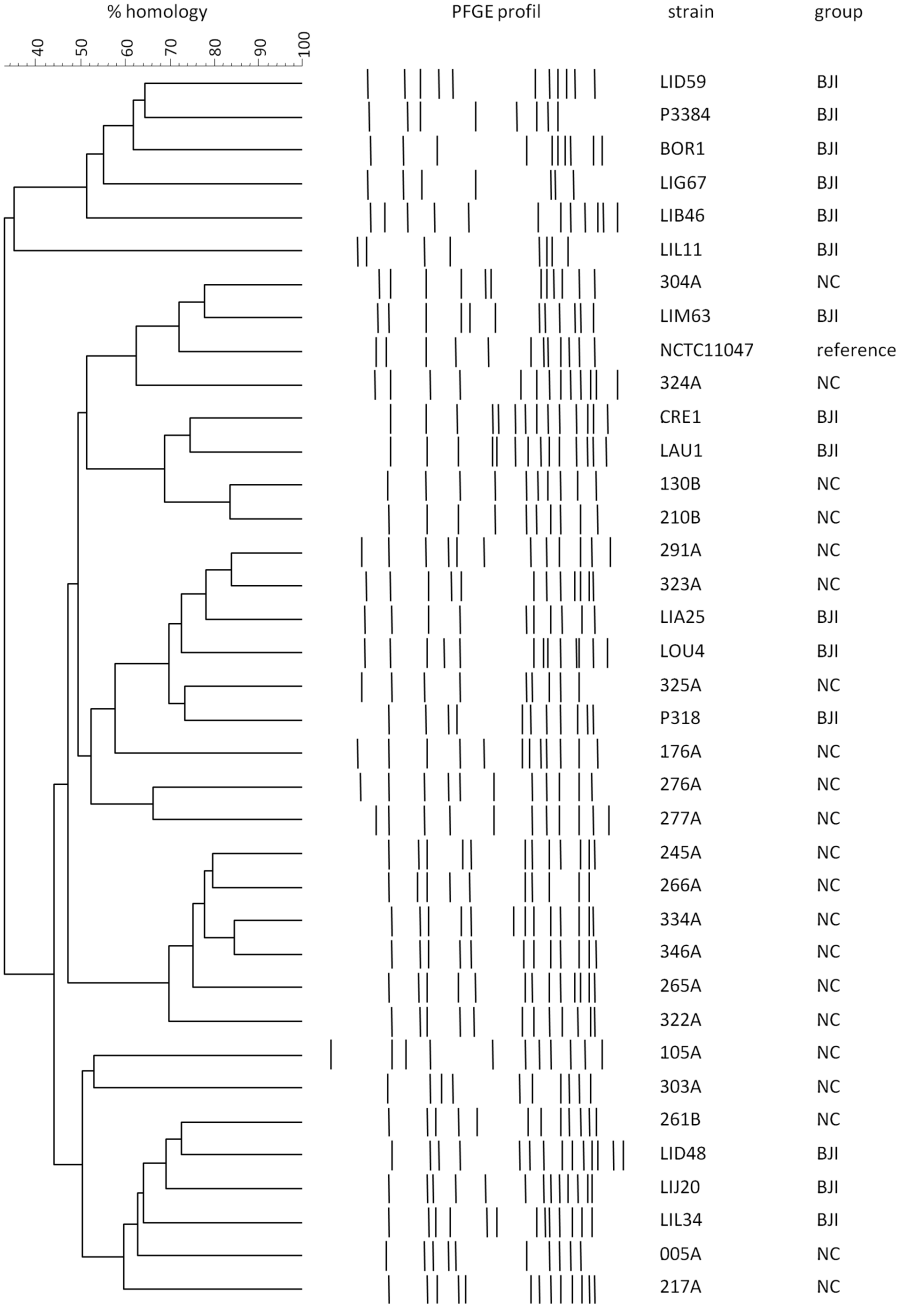
Pulsed-field gel electrophoresis (PFGE) pattern analysis. The number on horizontal lines indicates the percentage of homology by the Pearson correlation. BJI: Bone and joint infective strains; NC: Nasal carriage stains. One strain remained resistant to *Sma*I digestion and was assigned a specific non-restricted profile.

The 38 *S. epidermidis* isolates exhibited 34 *Sma*I PFGE types, demonstrating a high genetic diversity between the tested strains (fig. 6). One strain remained resistant to the *Sma*I restriction enzyme and was assigned a specific non-restricted profile.

## Discussion

Our study investigated whether internalization in osteoblasts and biofilm formation represented virulence factors that could discriminate *S. epidermidis* strains causing orthopedic device infections from colonizing ones. No differences were demonstrated between the two clinical populations (nasal versus orthopedic device infection strains) concerning their ability to invade MG63 osteoblastic cell lines. Similarly, biofilm formation, measured in its early and late phases by the Biofilm Ring test and crystal violet staining method, respectively, failed to discriminate these two isolate groups. Molecular typing of all strains by PFGE demonstrated a great diversity within the tested strains, which excluded the possibility that a low diversity was contributing to the absence of observed differences.

Some limitations of this study must be addressed. First, the selective use of gentamicin-susceptible isolates due to technical requirements to evaluate bone cells invasion using a gentamicin protection assay may have introduced a selection bias. Indeed, strains that may have acquired specific virulence factors associated with gentamicin resistance were excluded. However, we did not observe support for such a hypothesis in the published literature, so we consider that this bias likely does not alter our results. Second, based on the physiopathological model of *S. epidermidis* orthopedic device infection, colonizing strains from skin would have been more appropriate than from nares. Again, we did not identify any data that has demonstrated that colonizing *S. epidermidis* strains differed according to the site of carriage.

Several studies have attempted to identify features that distinguish invasive and pathogenic *S. epidermidis* strains from colonizing ones by mostly focusing on specific virulence factors. The IS256 mobile element has been epidemiologically related to *S. epidermidis* infecting isolates and proposed as a molecular marker for invasive strains [16], [17]. This insertion sequence has been identified in 26 out 32 prosthetic joint infection isolates, whereas only one of the commensal strains harbored it [17]. Moreover, Gu et al. reported that insertion of IS256 within the *agr* locus in clinical isolates decreased *agr* expression and consequently induced a global disruption of staphylococcal virulence factors that finally enhanced the success of *S. epidermidis* during infection of indwelling medical devices [16]. The role of the IS256 element in infection has also been attributed to its potential direct impact on biofilm formation due to its insertion within the poly-N-acetyl-glucosamine (PNAG)/polysaccharide intercellular adhesin (PIA) gene [18], which allows for the production of mature biofilms. Given that the production of PNAG/PIA is also regulated by the *ica*-operon genes, this operon has been proposed as a discriminating marker between *S. epidermidis* strains responsible for infection and colonization [19]. This marker was detected in 54 *S. epidermidis* strains collected from patients with bone and joint infections (81.5%), whereas only 17.4% of commensal isolates (n = 23) harbored this operon (p<0.01) [19]. Nevertheless, PNAG/PIA has been excluded as the unique pivotal factor for biofilm formation, and isolates lacking *ica* operon have been demonstrated to form biofilms through other adhesion proteins such as biofilm-associated protein (BAP) or accumulation-associated protein (AAP) [20]. More globally, if biofilms, and consequently most biofilm-associated virulence factors, participate in pathogenesis, they are also likely involved in maintaining species in a commensal state. For example, the importance of adhesins and proteins involved in the physiological biofilm formation (such as PNAG/PIA) during skin and mucous membrane colonization, where bacteria are subjected to significant mechanical forces, has been thoroughly demonstrated [21]. This finding could explain why numerous other studies have shown that invasive and commensal *S. epidermidis* strains could not be distinguished on the basis of their virulence factors [21]–[23], which supports the hypothesis of some authors of an accidental origin of infection from colonizing strains.

An unexpected insight from our study is the difference in bone cell invasion rates between the *S. epidermidis* and *S. aureus* strains. In 2007, Khalil et al. suggested a role for osteoblast invasion in the pathogenesis of *S. epidermidis* bone and joint infections based on the results from a limited number of clinical isolates (n = 4), which included only one bone-infective strain [12]. Here, we describe the first evaluation of the ability of a large collection of clinical *S. epidermidis* strains to invade bone cells. We demonstrated an extremely low internalization rate, 0.006% of the inocula, compared to 0.5 to 5%, which was classically observed for *S. aureus* in the same model. The particular nature of cell lines that are classically associated with the loss of some phenotypic characters led us to consider a possible “cell line effect” that may not reflect the *in vivo* reality [24]. Therefore, we repeated experiments on primary human osteoblasts, which yielded concordant results and confirmed the low ability of *S. epidermidis* to be internalized by cultured human osteoblasts in our model. As *S. aureus* adhesion to osteoblasts (via the staphylococcal fibronectin-binding protein (FnBP) interaction with cellular α5β1 integrin) had been shown as necessary and sufficient for its internalization [6], we explored this step of the osteoblast - *S. epidermidis* interaction. Our results demonstrated that *S. epidermidis* adhered to osteoblasts at an extremely low rate. Nevertheless, the flow cytometric-based approach only evaluates strong interactions, such as α5β1 integrin/FnBP. As FnBP are absent in *S. epidermidis*, the mechanisms of cell adhesion may be different. However, this does not challenge the results observed in the *S. epidermidis* internalization model, as the method quantifying bone cell invasion is independent of interaction type. Our data also suggest that internalization within osteoblasts is a less important pathophysiological mechanism for *S. epidermidis* orthopedic device infections than for *S. aureus*. Similarly, this mechanism seems to be more important in other clinical contexts involving *S. epidermidis*, such as bovine mastitis or urinary or endovascular infections. Indeed, several studies have demonstrated a high *S. epidermidis* internalization rate within endothelial [25], urothelial [26] or bovine mammary epithelial cells [27], [28]. Given that *S. epidermidis* is a universal commensal that expresses virulence factors that do not necessarily differ depending on the site of infection, it would be interesting to evaluate strains that are highly internalized in epithelial mammary cells in the context of our osteoblast infection model and vice versa. This proposal is supported by the Khalil et al. study, which demonstrated that a strain isolated from peritonitis was more internalized by osteoblasts that the bone-infective strain [12]. Nevertheless, it may be misleading to compare data from different protocols obtained with different cell lines and strains. A cross-comparison of the strains used in both models is ongoing and should reveal whether the phenomenon is dependent on the cell or bacterial type.

### Conclusions

The abilities of *S. epidermidis* to internalize in osteoblasts and to form biofilms failed to distinguish *S. epidermidis* strains isolated from infected orthopedic devices from nasal carriage isolates. These results support the hypothesis that *S. epidermidis* infections are an accidental event arising from the commensal flora, which colonize and infect strains that are clustered in a single microbiological entity. Given that a sub-group of patients with a higher risk of surgical site infection could not be detected based on the virulence of their *S. epidermidis* colonizing strains, only strict surgical asepsy may be able to reduce the incidence of *S. epidermidis* orthopedic device infections.

Our study provides the first assessment of the interaction between osteoblasts and a collection of clinical *S. epidermidis* strains responsible for bone and joint infections. Given our results obtained in our *ex vivo* models, internalization of *S. epidermidis* in human osteoblasts is not a likely major pathophysiological mechanism in orthopedic device infections, contrary to what is observed in other clinical situations, such as bovine mastitis or endovascular infections, or with other pathogens, such as *S. aureus*.
